# Rationale and development of an e-health application to deliver patient-centered care during treatment for recently diagnosed multiple myeloma patients: pilot study of the MM E-coach

**DOI:** 10.1186/s40814-023-01307-0

**Published:** 2023-05-20

**Authors:** Paul Geerts, Job Eijsink, Albine Moser, Peter ter Horst, Cornelis Boersma, Maarten Postma

**Affiliations:** 1 Department of Internal Medicine, Isala Klinieken, Zwolle, Netherlands; 2 Division of Hematology, Department of Internal Medicine, Research School GROW, Maastricht University Medical Centre, Maastricht, Netherlands; 3 Department of Family Medicine, Research School CAPHRI, Maastricht University, Maastricht, Netherlands; 4 Department of Clinical Pharmacy, Isala Klinieken, Zwolle, Netherlands; 5 Department of Health Sciences, University Medical Centre Groningen, Groningen, Netherlands; 6 Zuyd University of Applied Sciences, Heerlen, Netherlands; 7 Faculty of Management Sciences, Open University, Heerlen, Netherlands; 8 Unit of Pharmacotherapy, Epidemiology & Economics, Groningen Research Institute Pharmacy, University of Groningen, Groningen, Netherlands; 9 Department of Economics, Econometrics and Finance, Faculty of Economics and Business, University of Groningen, Groningen, Netherlands

**Keywords:** Myeloma, Supportive care, E-health, E-coach, Medication

## Abstract

**Background:**

Patients with multiple myeloma (MM) increasingly face complicated treatment regimens. E-health may support patients and healthcare providers in enhancing a patient-centered healthcare approach. Therefore, we aimed to develop a patient-centered multi-modality e-health application, to assess the application for usability and end-user experiences.

**Methods:**

The application was developed following an iterative “action-based” methodology using the design thinking approach. Key end users participated, and relevant stakeholders were consulted in the development process. First, the care pathway was evaluated, the focus of development was determined, and a solution ideated during recurring multidisciplinary meetings. Second, a prototype was tested and improved. Third, a subsequent prototype was evaluated during a pilot study with patients and healthcare professionals on usability, usage, and experiences.

**Results:**

The multi-modality application, named the “MM E-coach,” consisted of a newly developed medication module, patient-reported outcome (PRO) questionnaire assessments, a messaging service, alerts, information provision, and a personal care plan. The median system usability score was 60 on a scale of 0–100. Patients appreciated the medication overview, healthcare professionals appreciated the outpatient clinic preparation module, and both appreciated the messaging service. Additional recommendations for improvement mostly revolved around the flexibility of functionalities and look and feel of the application.

**Conclusions:**

The MM E-coach has the potential to provide patient-centered care by supporting patients and caregivers during MM treatment and is a promising application to be implemented in the MM care pathway. A randomized clinical trial was initiated to study its clinical effectiveness.

**Supplementary Information:**

The online version contains supplementary material available at 10.1186/s40814-023-01307-0.

## Key messages regarding feasibility

- What uncertainties existed regarding the feasibility? We aimed to develop a novel e-health product, without being sure how this product would most optimally align with (a) the patient needs and (b) the healthcare providers work process. Subsequently, we did not know what the optimal design would be for reaching as much impact as possible.
- What are the key feasibility findings?
  - Most of the modules that we developed proved an added value for a large part of the patients and healthcare professionals. However, there was never a 100% coverage, and we learned that usage needed to be individualized.
  - We learned about very many “look and feel” issues, which helped us in improving the interface and easiness of use of the application.
  - For final implementation in practice, a link between the application and the hospital systems was required.
- What are the implications of the feasibility findings for the design of the main study? Finally, we were not yet able to make the link as described above. The most important implication, besides many small design changes, was to hire a research nurse to replace the actions that the link would ideally take care of in practice. This way we were able to start the clinical trial while waiting for the expected resources that will provide the link at a later moment (this is currently still planned in the future by the developer, awaiting certain updates).

## Background

Multiple myeloma (MM) is a blood cancer of monoclonal plasma cells that accumulate in the bone marrow and may be complicated by organ dysfunction, such as hypercalcemia, renal insufficiency, anemia, and bone destruction. It accounts for 1% of neoplastic diseases and is the second most common hematological malignancy [1]. Over past decades, the survival of patients with MM has improved due to new treatments [2]. Current effective regimens combine three or four anti-myeloma drugs [3–5]. These drugs are applied in complicated treatment schedules, with concomitant drugs to prevent or treat infection, thrombosis, nausea, and pain. Such treatment schedules may be difficult to understand for patients [6]. Furthermore, these treatments have been investigated in randomized clinical trials, and most patients in the real-world setting are not considered eligible for such trials [7]. Therefore, experts agree that the applications of these treatments in the real-world setting may be limited due to various patient-, treatment-, and disease-related factors. Instead, they recommend patient-centered healthcare delivery [8]. Many definitions and models for patient-centeredness exist [9]. The WHO proposed a strategic framework aiming to deliver person-centered, integrated, and proactive healthcare services [10].

To deliver such care to patients with MM receiving active treatment, the use of electronic health (e-health) innovations may be considered. e-health refers to the broad use of health information and communication technologies and networks to enhance patient-centered care delivery. It has the potential to improve patient-provider communication, to enhance symptom and toxicity assessments, to optimize patient engagement, and to facilitate care access [11]. Although e-health applications for cancer patients are numerous, reports on their application to patients with MM are limited [12]. Examples of patient-centered e-health functions or modules in other types of cancer may include the following: first, PRO assessments, including patient-reported outcome and experience measures (PROMs and PREMs) [13–15]; second, communication systems between patients and/or healthcare providers [16]; third, modules may include education, aiming to improve patients’ knowledge of their condition [14, 15]; and finally, intervention modules aiming to influence behavior or empower patients, such as applications aiming to improve medication adherence [17, 18]. To deliver person-centered, integrated, and proactive care, care pathways have been introduced. An e-health application for the MM care pathway would ideally align with both patients’ daily lives and with healthcare providers’ workflow [19]. This requires, besides a needs assessment, an iterative development process with end users to continuously check the intended effect and process [20–23].

To address the care needs for patients with MM, we designed a project with the overall aim to improve care for patients with MM, collaborating with all relevant stakeholders. This project was based on a value-based healthcare (VBHC) methodology, aiming to improve outcomes for patients with MM [24]. The VBHC project consisted of developing a new care pathway, an outcome set for patients with MM and an e-health application based on the needs and preferences of patients with MM and their healthcare providers. In the current study, we focus on the development of the e-health application by an iterative process with the participation of all relevant stakeholders. At that time, care delivery — especially with regard to the information and communication technologies — was fairly fragmented from the patients’ perspective, and a multi-modality application was expected to support delivering patient-centered care.

The aim of the current study is twofold as follows: first, developing a multi-modality e-health application for patients with MM and their healthcare providers, aligning with the new MM care pathway, and second, assessing the resulting application for usability and end-user experiences.

## Materials and methods

**Fig. 1:**
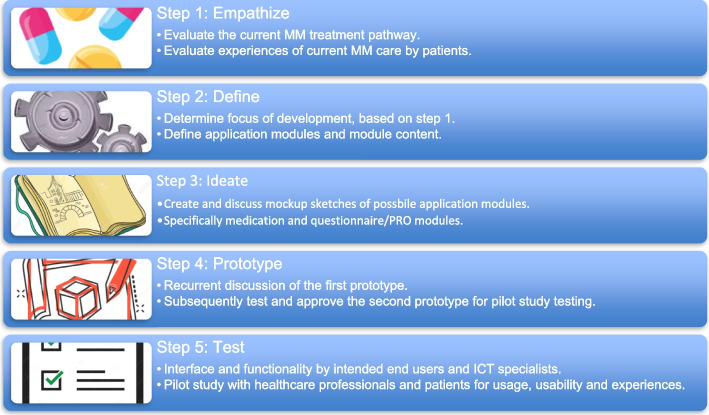
The five-step development process of design thinking

The study used an iterative “action-based” methodology and followed the five-step process of the design thinking approach (i.e., empathize, define, ideate, prototype, and test) (Fig. 1) [20, 25]. The study was performed in recurrent development team meetings, with the final two steps during a pilot study. Additional meetings to design a new MM care pathway and outcome set occurred parallel to the development meetings. First, we describe the development steps 1 to 3, reporting about the meetings. Then, we describe the development and testing of the first and second prototypes, reporting about the pilot study.

**Fig. 2:**
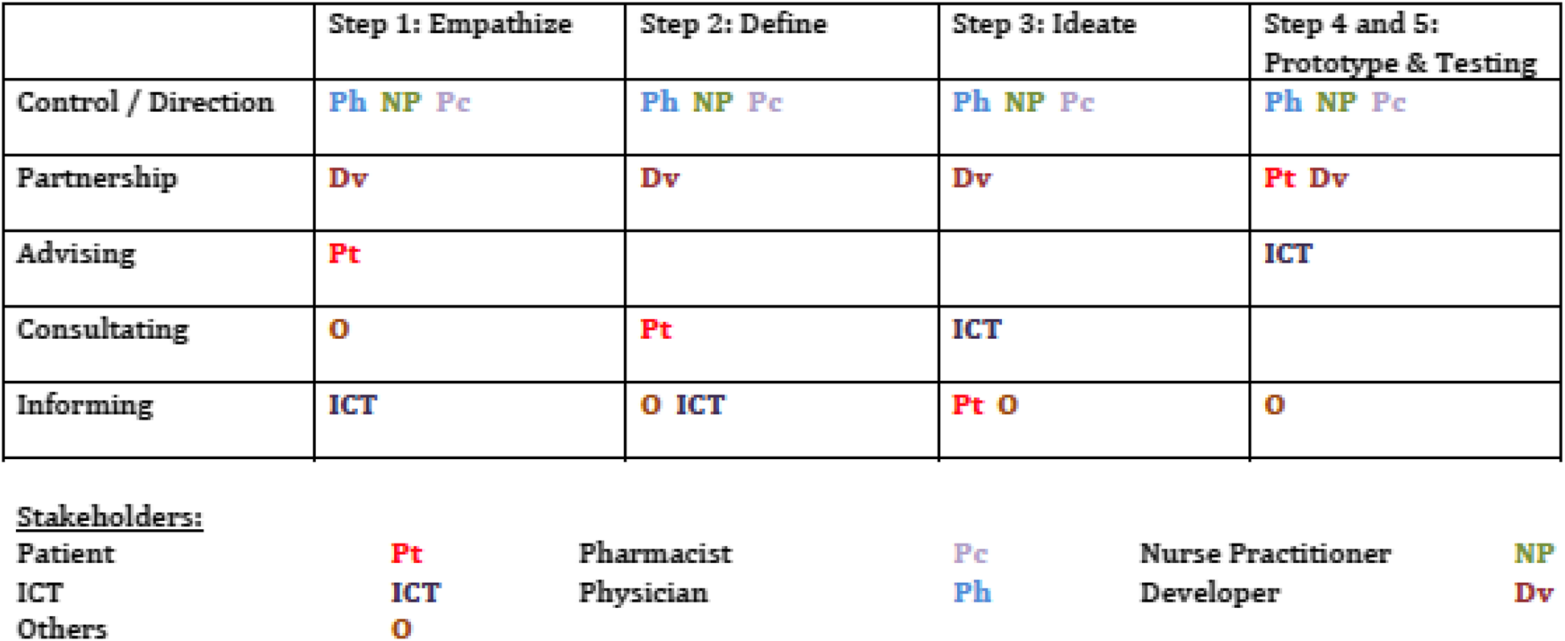
Stakeholder participation matrix. The columns represent the five steps of the design thinking approach (steps 4 and 5 are depicted in one column), and the rows represent the five ascending levels of stakeholder participation

Throughout the study, the intended end users (patients, hematologists, and nurse practitioners) and clinical pharmacists actively participated in the application design (co-creation), and additional stakeholders were consulted in a dynamic development process [26]. Fig. 2provides an overview of participation for the most relevant stakeholders at each development step [27]. Sananet Care B.V, a Dutch e-hHalth development company, performed technical development support. Amgen B.V., a pharmaceutical company, sponsored the development company with an unrestricted grant and was not involved in the actual development. Isala Klinieken, the hospital where the study was performed, provided a study nurse free of charge to the development team and was not otherwise involved in the actual development.

### Setting

The study was performed in a large nonacademic hospital in the Netherlands, treating about 50 patients with newly diagnosed MM each year. In the MM care pathway, seven hematologists and four nurse practitioners provided care, in collaboration with hospital pharmacists, clinical ward professionals, and outpatient daycare clinic professionals. All treatment schedules were applied, including autologous stem cell transplantation, following MM treatment guidelines [28]. The hematologist and nurse practitioner performed diagnostics and pre-treatment evaluation and provided information to patients. Oral medication was provided by the pharmacy directly to the patients with instructions. Subcutaneous or intravenous medication was prepared by the pharmacy, distributed to the daycare clinic or clinical ward, and administered by oncology nurses. Oncology nurses also administered some medications at home, mostly subcutaneous injections. Before each subsequent treatment course, the hematologist performed an outpatient clinic evaluation, including laboratory evaluation and assessment of symptoms or side effects. The nurse practitioner performed periodic well-being assessments and provided supportive care. Between visits, patients could e-mail or call the nurse practitioner or hematologist. For acute health issues, patients were referred to the emergency department or for an outpatient clinic visit.

### Steps 1 to 3: Empathize, define, and ideate

#### Participants

At the start of the project, all relevant stakeholders involved in the MM care pathway were identified. Representatives of each stakeholder category were invited to participate in the development team by purposive sampling, based on motivation to participate in this project. The development team consisted of two physicians, two nurse practitioners, three pharmacists, a manager, a secretary, a data manager, a quality and innovation department delegate, an information and communication technology (ICT) delegate, two developer employees, and a representative of the sponsor. Additionally, three patients with MM and their spouses were invited by convenience sampling.

#### Procedures

The first three steps were performed over a 1-year period during six development meetings. The development team attended all meetings, and the patients and their spouses attended the third meeting. Between and following the meetings, smaller subgroup meetings were organized to elaborate on specific subjects, if needed. The development team also attended the parallel care pathway and outcome set development meetings.

At step 1, empathize, the following information was discussed with the development team: first, a detailed description of the current MM care pathway, and second, results of a survey among 18 patients with MM, exploring the current experience with care delivery and the needs for improvement. The team agreed on the ideal care pathway by consensus, aiming to provide more patient-centered care. It included integrating an e-health application.

At step 2, define, joint aims and targets were set to determine the focus of development for the e-health application. At this point, the patients and their spouses participated. The team agreed on the desired application modules and content, aligning with the care pathway. The content was also based on the MM outcome set, which was defined in parallel meetings by discussing known clinical and patient reported outcomes and a second survey, evaluating current symptoms among the 18 patients with MM.

At step 3, ideate, mockup display sketches of the intended application modules were recurrently performed, discussed, and optimized among the development team. In this phase, patients were involved by informing and the development team used their input from the questionnaires and the earlier meeting.

#### Data and analysis

The project manager gathered the data of the development meetings using detailed written summaries. For each subsequent meeting, the summary of the previous meeting was member-checked by the attendees. The summaries were analyzed with content analysis [29].

### Steps 4 and 5: Prototype and test

#### Participants

Four healthcare professionals from the development team (one physician, two nurse practitioners, and one pharmacist) and ICT specialists tested the first prototype. The second prototype was tested in a pilot study, including patients and healthcare professionals. Hematologists, nurse practitioners, and pharmacists, including those participating in the development team, were approached to participate using purposive sampling. They were involved in MM care and open minded towards innovations. Patients were recruited from the outpatient clinic of the participating hematologists by convenience sampling. Patients 18 years and older receiving treatment for MM according to the International Myeloma Working Group (IMWG) criteria were eligible for inclusion [30]. Exclusion criteria were mental or physical illness requiring clinical admission, lack of Wi-Fi Internet access, or the ability to operate a tablet and inability to understand the Dutch language. We aimed to include two hematologists, one pharmacist, four nurse practitioners and 20 patients.

#### Procedures

Prototyping and testing were performed in an iterative manner. The first prototype was assessed for content, interface, comprehensibility, functionality/navigation, and usefulness for practice on various devices with test patient cases. Requirements for further development were then provided and performed by the developer. Subsequently, additional verification was performed on these improvements, and the development team approved the second prototype for the pilot study.

The second prototype was pilot tested from June 2020 until August 2020, evaluating usage, usability, and experiences. The healthcare providers received training from the developer of the application. Patients were asked for informed consent by the hematologist. Then, a nurse practitioner attended participating patients at home for a short introduction of the study and the application. The nurse practitioner also handed the patient a tablet for the study period with access to the e-health application and a video consultation application. Patients received a unique username and password and filled out the first periodic PRO questionnaire during the visit. Subsequently, patients used the application for 8 weeks, during which care was provided following the new care pathway, including the application. This also included more intensive collaboration between the hematology and pharmacy departments.

The results of the pilot study were evaluated during a 2-h session with the development team, with the exception of patient representatives. Due to the COVID-19 pandemic, patients could not attend the evaluation meeting. Patients were sent the results by post, including a form to provide additional feedback and the possibility to elaborate by phone. All patients provided written feedback, and two patients were additionally interviewed by phone. The development team combined the patient feedback with the other feedback and made a list of required improvements for further development.

#### Data and analysis

The participants provided remarks point by point for each module of the first prototype to the project manager, who made a structured report of all gathered remarks. The report was member checked by all participants and subsequently analyzed with content analysis.

At the end of the pilot study, usability was evaluated with patients with the system usability scale (SUS) [31]. This is a validated 10-item questionnaire covering learnability and satisfaction [32]. The score was calculated on a scale of 0–100, where 100 reflects highest usability. Usability testing helps us to uncover problems, discover opportunities, and learn about users. A mean score of 70 is associated with good user-friendliness [33]. Healthcare professionals received a self-developed questionnaire addressing the added value of the application (Additional file 3: Appendix A). Additionally, the developer collected the data for usage activities on the application. At the end of the pilot study, an overview was provided including the usage frequencies of all modules. Data were analyzed by descriptive statistics (medians and frequencies) using SPSS (IBM Corp SPSS Statistics, version 26. Armonk, NY, USA).

Besides usability and usage, qualitative experiences with the application were evaluated. During the pilot study, the participating healthcare professionals filled out “case forms” (Additional file 3: Appendix B). The professional indicated if and how care provision using the application was different from usual care. The case forms were discussed during weekly meetings with the participating healthcare professionals, summarized, and provided with comments. Furthermore, patients and healthcare professionals were asked to submit any relevant experiences with the application or recommendations to improve it during the 8-week study period. During the final study evaluation, minutes were written, summarizing the discussion and the final recommendation of each suggested improvement. The phone interviews with patients were summarized with notes. All qualitative data were collected by the project manager and analyzed using content analysis. This information was summarized into one list with recommendations.

#### Trustworthiness

To secure credibility, the empathize step was performed for prolonged engagement and the iterative process for persistent observation. We included researchers with different backgrounds and various stakeholders in the development team (peer debriefing) and used the mixed-methods methodology for different perspectives (data and source triangulation). The evaluation with feedback at the end of the study provided a member check of the final recommendations. The meeting and evaluation summaries were stored and available for review afterwards. To secure transferability, the development team and study setting were extensively described, and example figures of the development steps were included in the results.

#### Ethics

The Medical Research Ethics Committee (METC) of Isala Klinieken approved the study, waiving the requirement for obtaining informed consent (local METC number 200324).

## Results

First, we provide a summary of the results of development steps 1 to 3. Then, we describe the development and testing of the first and second prototypes in detail.

### Steps 1 to 3: Empathize, define, and ideate

At the first step, empathize, the care pathway was found to consist of a diagnostic, treatment, and follow-up phase. With regard to the e-health application, the development team focused on the treatment phase. Here, a patient encountered many medical investigations, visits, and medication prescriptions. This required elaborate logistics and information exchange that was mostly organized following hospital logistics. Most actions took place in the hospital at the outpatient clinic, the daycare clinic, and the pharmacy. Usually, patients only attended the clinical ward when experiencing severe side effects or complications. A new care pathway integrated several services and was designed more at the convenience of patients. The outcome set included medical outcomes, such as overall and progression-free survival. Furthermore, it included patient-reported outcomes (PROMS and PREMS), for example, addressing quality of life, pain, common treatment complications, and information and decision-making.

At development step 2, define, the team agreed on six aims and targets that were used as starting principles for the design and development of the e-health application (Table 1). Based on these principles, the application modules were defined (Table 2).

**Table 1:** Aims and targets

Aim 1: Providing treatment and support at home when possible and in accordance with the wishes of patients
Aim 2: Providing personalized medication overview and support
Aim 3: Optimizing therapy adherence, including side effect management
Aim 4: Optimizing quality of life
Aim 5: Optimizing patient safety
Aim 6: Improving progression-free survival

**Table 2:** MM E-coach module overview

Module	Description	Use
Medication	1. Information and overview of MM medication dose, frequency, and time 2. Therapy adherence tool with reminders and medication dose registration	Continuously during treatment
Outpatient visit preparation	Practical information. Questionnaire with pain, fatigue, shortness of breath, frailty, and neuropathy. An open question is as follows: “What do you want to discuss with your hematologist/nurse?”	One week before outpatient clinic visit
Periodic assessment	Patient-reported outcomes	Every 3 months
Ad hoc complaint	Ad hoc complaint form with automated advice algorithm	Continuously available
Messaging service	Bilateral messaging service with healthcare provider	Continuously available, reply during weekdays
Alerts	Algorithm detecting severe complaints or side effects based on “red flag” thresholds, warning patient and healthcare provider to engage (immediate) contact	Continuously available for patients, check and reply by provider during weekdays
Information	Information about MM, linked to healthcare provider website	Continuously available
Personal care plan	Overview of disease activity in time	Continuously available

At step 3, the application modules were ideated. For example, a medication module interface was recurrently reviewed (Additional file 1: Fig. S1). Furthermore, PRO questionnaire algorithms were built, asking tailored follow-up questions depending on earlier answers. For example, when patients indicated not having pain, no follow-up about pain would be asked.

### Steps 4 and 5: Prototype and test iteration 1

The first prototype consisted of all modules as described above, albeit in a simple test layout. Testing generated 81 feedback items with requirements for improvement. These are summarized by category in Table 3. Most concerned small interface or functional items, such as “Two icons were depicted on top of each other instead of next to each other.” However, some items concerned the algorithm behind the application (e.g., “When I answer that I have taken medication for pain, it does not ask me what medication.”).

**Table 3:** Feedback for improvement of prototype 1, categorized

Category
Bugs (functionality and interface)
Differences between web-based and mobile device application version
Missing functionalities
Navigation issues
Language/textual problems
Missing content
Error in content (algorithm and information)

### Steps 4 and 5: Prototype and test iteration 2

**Fig. 3:**
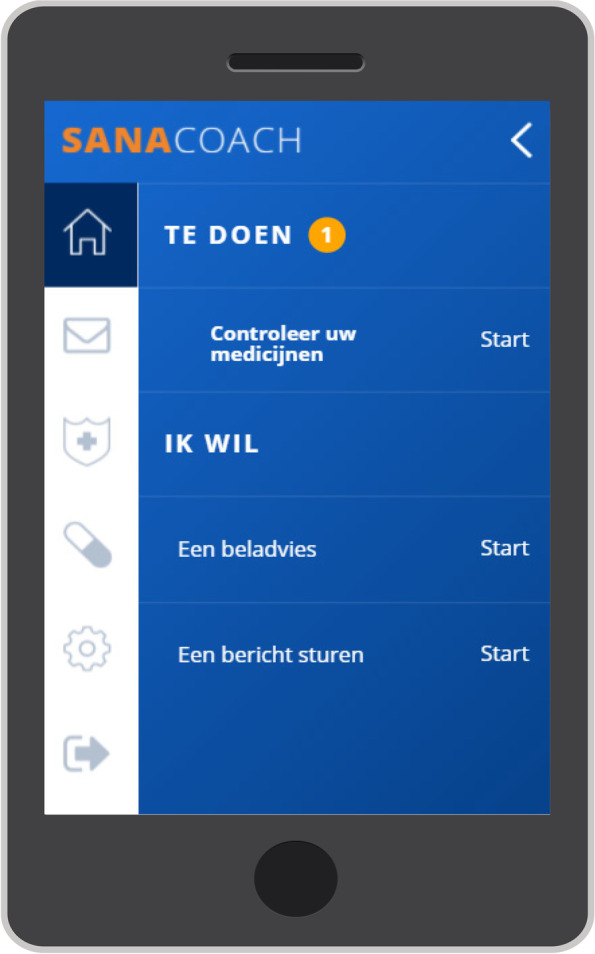
MM E-coach index for patients. The text lines represent the following: “TO DO,” “Check your medication,” “I WANT,” “A telephone advice,” and “To send a message”

The second prototype was a web-based application called the “MM E-coach” that was available on multiple devices, including mobile devices. It required a personal login protected by a secure link with two-way verification. The patient version was used as a stand-alone program (Fig. 3, index overview). The healthcare provider version was also partially integrated in the electronic health record program (HiX 6.1, Chipsoft). Patients and healthcare providers received a manual and on-demand technical support from the development company. The MM E-coach included eight modules (Table 1).

- Module 1: Medication module. It provides a daily overview of prescribed medication (Additional file 1: Fig. S2). For each available treatment course, a template was made, including the anti-myeloma medication and concomitant medication. The patient can register medication intake (Additional file 1: Fig. S3). The healthcare provider can view a daily, weekly, or therapy course medication overview, including the registration of the patient to assess therapy adherence.
- Module 2: Outpatient visit preparation. The patient is requested to complete a short questionnaire 1 week before each visit, including a blank space to inform the healthcare provider what the patient would like to discuss (Additional file 1: Fig. S4). This questionnaire was based on the most important items determined during discussion of the symptom questionnaire with patients and healthcare providers. The patient and healthcare provider can evaluate short-term well-being and decide what may (not) be discussed during the consultation.
- Module 3: Periodic assessment. The patient is requested to complete a more elaborate periodic questionnaire, including PROMS and PREMS (Additional file 1: Fig. S5). This questionnaire was based on the MM outcome set that included, among other aspects, validated questionnaires assessing quality of life (EORTC QLQ-30 [34] and EQ-5D-5L [35]) and therapy adherence (MARS-5 [36]). The frequency may vary based on local preferences. The patient and healthcare provider can evaluate long-term well-being, aiming to comply with personal treatment goals.
- Module 4: Ad hoc complaint. It allows a patient to report a complaint or side effect at any time (Additional file 1: Fig. S6). An algorithm with thresholds (settings according to local protocol) provides the patient with advice or notifies the patient to (immediately) contact the healthcare provider.
- Module 5: Messaging service. It enables patients and healthcare providers to ask and reply to questions at a moment of convenience. A disclaimer indicates the usual time to a reply from the healthcare professional (in our clinic < 24 h during weekdays) and advises patients to directly contact the clinic for emergency situations.
- Module 6: Alerts. It notifies the patient to (immediately) contact the healthcare provider, based on thresholds for the periodic check and visit questionnaire items (Additional file 1: Fig. S7). It also generates an alert for the healthcare providers, appearing in the messaging service inbox with a red icon.
- Module 7: Information. It provides a patient with information about the disease, therapy, or general information (e.g., with treatment booklets or information about the clinic).
- Module 8: Personal care plan. The patient and healthcare provider can summarize a personal care plan, including treatment goals.

### Pilot study characteristics

In the pilot study, 20 patients, two hematologists, four nurse practitioners, and one pharmacist were included. Two patients died early, which was unrelated to the use of the MM E-coach. Their data were incomplete and could not be evaluated. The median age of the remaining 18 patients was 63 years (range: 47 to 84 years). Fifteen patients were male, and three patients were female. All patients received a second or later line of anti-myeloma therapy.

### Pilot study usability and usage

At the end of the study, the median SUS score by patients was 60. At that time, 83% of patients indicated being willing to continue using the MM E-coach. All 18 patients completed the first outpatient visit preparation questionnaire. The periodic PRO questionnaire completion percentages were 94% at the start of the study and 72% at the end of the study. Fifteen patients (83%) used the messaging service, generating 101 messages (nearly seven messages per patient). The healthcare providers responded with 85 messages, mostly by nurse practitioners. Five patients used the ad hoc complaint module once. One patient was advised to immediately contact the healthcare provider by the alert module. The patients registered 47% of the expected medication intake.

Six healthcare providers completed the evaluation questionnaire. All agreed that the MM E-coach helped them in performing their daily clinical work and provided insight into the well-being of patients. All thought the MM E-coach would fit into future healthcare. While the other five participants agreed, one participant disagreed that using the MM E-coach was more efficient than usual care.

### Pilot study qualitative evaluation

A summary of the evaluation is provided in Additional file 2: Table S1, and some highlights are described here. The patients appreciated the medication overview and the messaging service, with comments such as “It is easy to see whether I have taken my medication,” “I appreciate the daily medication overview,” and “Contacting is flexible and accessible.” However, they also experienced various practical, technical, and flexibility problems, with comments such as “I would like a function that reminds me when I have forgotten to take my medication,” “I would like to register taking multiple medications at one time instead of registering each medication separately,” and “I have to log in every time and would prefer it to be automatically.”

The healthcare professionals also appreciated the messaging service and acknowledged the added value of the outpatient clinic preparation module, with comments such as “An accessible way getting into contact with patients” and “The module visualizes highlights and works properly.” They also indicated several practical and technical problems, with comments such as “It takes a lot of time to enter the medication in the MM E-coach, it should feed automatically” and “The questionnaire outcomes are not so clear, I recommend a dashboard functionality.”

Analysis of the case forms illustrated the potential of the MM E-coach in clinical practice. For example, a patient indicated shortness of breath during treatment, due to which he was assessed earlier than scheduled on the outpatient clinic. It was subsequently decided to admit the patient to the clinical ward for treatment. The healthcare providers expected that with regular care, the patient would probably have been admitted to the emergency department, and clinical ward admission would have lasted longer, possible also compromising myeloma treatment.

### List of requirements

Following the evaluation of the outcomes with the development team, a list was formulated with 16 additional improvements to make the MM E-coach suitable for use in standard clinical practice (Table 4). Most concerned is the medication module. As the research team noticed a need to prioritize possible improvements during the evaluation meeting, they were ranked as “need to have,” “nice to have,” or “not necessary.”

**Table 4:** List of requirements

Module	User	Requirement
Medication	Patient	Enable registering medication at a later moment
Medication	Patient	Introduce optional push message alert, as reminder for medication
Medication	Patient	Optimize medication registration, including the following: •Early registration (when taken earlier) •Late registration (when taken but not registered)
Medication	Both	Solve bugs in medication overview and add user-friendly functionalities
Medication	Both	View medication in a separate tab
Medication	Both	Automated medication feed from EHR
Medication	Both	Add additional medication information
Medication	Both	Distinguish standard and “as-needed” medication more clearly
Medication	Professional	Optimize medication adjustments
Medication	Professional	Change search engine when adding new medication, making it more intuitive
Messaging	Both	Change the message order
Alerts	Professional	Optimize alert triggers
Periodic assessment	Both	Create a dashboard to view PROs
Visit preparation	Professional	Automated appointment feed from EHR with questionnaire trigger
-	Professional	Add functionality “Burden of disease registry”
-	Patient	Several changes to the user manual for patients

*Abbreviations*: *EHR* Electronic health record, *PRO* Patient-reported outcome

## Discussion

In this study, we developed a multi-modality e-health application for patients with MM and their healthcare providers—the MM E-coach. The MM E-coach was developed together with the intended end users and several relevant stakeholders. The MM E-coach consists of eight modules, including a novel medication module. We assessed the MM E-coach for usability, usage, and experiences and formulated additional requirements before actual use in practice. The median system usability score was 60 on a scale of 0–100. The patients acknowledged the medication overview and the messaging service as an added value, compared to usual care. The healthcare providers acknowledged the outpatient clinic preparation module and being able to act early on patient-reported symptoms or side effects. At the same time, the study participants provided several useful recommendations to improve the MM E-coach.

This study was part of a VBHC project with the overall aim to improve the quality of MM care [24]. We intended to improve quality of care from the perspective of the quadruple aim and the WHO framework on integrated people-centered health services, leading to a design with participation of patients and healthcare professionals [10, 37]. Consequently, the MM E-coach addresses the three components of the WHO framework [10].

First, the MM E-coach contributes providing patient-centered care. The iterative, participatory development process informed the development team about “what matters” to patients. This resulted in functionalities that may empower patients to receive care based on their needs and individual situation. For example, the outpatient visit preparation module allows the patient to inform the professional beforehand the consultation, towards treatment goals and life questions. This may individualize the consultation and create more awareness of what matters most to the patient [38, 39]. Despite this approach, patients evaluated the second prototype with a suboptimal SUS score. The patients in our study received prior lines of therapy earlier and were therefore “experienced” patients that already had developed their individual coping mechanisms with the treatment period. Therefore, instead of indicating their disapproval of the MM E-coach, their extensive recommendations for improvement indicate that they see possibilities for future use, including for patients receiving later lines of therapy. Furthermore, besides providing patient-centered care on an individual level, aggregated patient data may be used in the future for patient-centered care for groups of patients. A possible need for such aggregated patient-based information is exemplified by the “PatientsLikeMe” initiative [40].

Second, the MM E-coach contributes to providing integrated care, although future steps may be needed to optimize this aspect. Current healthcare delivery is mostly organized around one specific system and/or specific disease, instead of being organized around the needs of their users [10]. In a similar way, the MM E-coach was designed to support patients receiving therapy for MM and healthcare professionals supporting these patients. In the past years, only for cancer already numerous e-health applications have been developed [41]. Future improvement or development of e-health applications, including the MM E-coach, may benefit from both generic access as well as flexibility, allowing individualized use based on the patients’ needs and situation. For example, this may require a generic application, usable for any patient, with disease specific “add-ons.” In turn, the MM E-coach would need to be compatible with and be accessible through such a generic application. Similarly, the disease-specific add-ons, including the MM E-coach, would need to be frequently updated, based on future developments in myeloma treatment and hospital care.

Third, the MM E-coach contributes to proactive care, which includes monitoring and easy and accessible communication. The messaging module provides patients with an accessible method to get in touch with the healthcare providers. Furthermore, the several questionnaire and report modules, in combination with advices and alerts, contribute to timely interventions by healthcare professionals, based on patients’ information. This may result in cost reduction and health improvement, for example, by less emergency room visits and improved quality of life, such has been shown in other cancer patient populations [42]. Additionally, following optimizing the medication module based on the recommended improvements, medication compliance and knowledge may be improved. Evaluation of these outcomes is part of the current randomized controlled trial evaluating the MM E-coach (EudraCT 2020–005,267-31).

### Methodological reflection

A strength of this study is the contextual, iterative, and participatory development process, integrating an e-health application in a revised MM care pathway. The main goal of this method is to close the gap between development and implementation, aiming to reach more clinical or societal impact of a healthcare innovation [20, 21, 23, 43]. An important aspect was the participation of the most important stakeholders at different levels during the development process, as depicted in the participation matrix in Fig. 2. Arnstein divided participation into eight levels, and nowadays, it is usually divided into five levels: informing, consultation, advising, partnership, and control [27, 44]. Patients and professionals were included in our development team as the intended end users of the application. The professionals primarily controlled the development process, and the participation of patients varied, up to a partnership role in the pilot study. This active participation is also called “co-creation.” [26] In future e-health development, including improving the MM E-coach, the degree of stakeholder participation should be determined for each design step and might differ from consultation to a full co-creative role (partnership). By using a participation matrix, participation can be negotiated, evaluated, and improved [27].

An additional method that may further increase the chance of widespread implementation and societal impact is business modeling [21, 22, 45, 46]. This methodology addresses sustainable implementation, maintenance, and governance of applications, increasing the chance of long-term use and impact. Before actual implementation, a business case discussion is planned with the developer and healthcare insurance representatives.

### Future perspectives

Currently, one important “need to have” improvement has not been performed yet and is being worked on: the automated medication feed from EHR.

A digital application may not be suitable for every patient. Incidentally, patients do not have Internet access or may not be equipped to utilize and navigate on mobile devices [47, 48]. A future step may be evaluating and adapting the MM E-coach among patients with low digital and/or health skills and in different age groups.

Finally, awaiting future developments for a possible generic application, a possible future step for the MM E-coach is to include other care situations, such as palliative care, or other (hemato-oncologic) diseases.

## Conclusions

In this study, an iterative and participatory design method was used to develop a multi-modality e-health application, the MM E-coach. It has the potential to support patients and healthcare professionals during MM treatment. Promising among the modalities is a novel medication module. Following adjustments in line with the recommendations in this study, the MM E-coach is currently being evaluated in a randomized clinical trial. In the future, continuous evaluation and development are required to create a dynamic MM E-coach in line with MM care demands.

## Supplementary Information

**Additional file 1: ** **Fig. S1.** Mockup example of a medication overview for patients of one of the treatment schedules (Rd; lenalidomide/dexamethasone and co-medication). The text boxes next to the date display time by day or week; The columns represent medication type, medication dose, medication time and additional instructions. **Fig. S2.** Medication overview of one of the treatment schedules (KRd; carfilzomib/lenalidomide/dexamethasone and co-medication). The menu to the left is identical to figure 3; the columns to the right are comparable to figure S1. **Fig. S3.** Medication registration page. The menu to the left is comparable to figure 3. Each line represents one medication with information, for example acetylsalicylzuur translates to acetylsalicylic acid and the bold text to ‘dosing’, ‘aim’, ‘brand types’, ‘time of preference’, ‘side effects’, ‘additional remarks’. **Fig. S4.** Outpatient visit preparation questionnaire (not showing blank space). The menu to the left is comparable to figure 3 and marks ‘Outpatient clinic preparation’. The text contains an introduction asking patients what complaints or side effects they experience at that give moment and states that a patient can tick as many boxes as apply to that moment. Then every line represents one complaint, for example ‘Braken’ means ‘Vomiting’. **Fig. S5.** Periodic assessment, one question example. The menu to the left is comparable to figure 3 and marks ‘Periodic check’. The question translates to ‘How would you judge your health during the last week?’ and the answers range from ‘very bad’  to ‘excellent’. **Fig. S6.** Ad hoc complaint form completed, followed by advice. This displays the healthcare provide account, zooming in on one specific part of a questionnaire that was filled out by a patient. The second yellow alert line states the question ‘Do you experience vomiting for more than 24 hours’, as a follow-up question when the patient has ticked ‘vomiting’ in the list in figure S4. At the most right it states that the patient has answered ‘Nee’ (No). **Fig. S7.** Healthcare provider alert list, including one active alert. This displays the healthcare provider account, highlighting the ‘Interventielijst’ (List of interventions) that indicates a red alert  for a given patient at a given time. 

**Additional file 2: Table S1: **Summary of qualitative evaluation.

**Additional file 3: Appendix A.**Pilot study questionnaire for healthcare professionals. The questionnaire was originally used in Dutch language and thisversion is a non-validated translation.**AppendixB.**Example of an empty case form. This version is a non-validated translation for purpose of the readers’ understanding.

## Data Availability

The datasets used and analyzed during the current study are available from the corresponding author on reasonable request.

## Abbreviations

COVID-19: Coronavirus disease 2019
e-health: Electronic health
ICT: Information and communication technology
IMID: Immunomodulatory drug
IMWG: International Myeloma Working Group
METC: Medical Research Ethics Committee
MM: Multiple myeloma
PREM: Patient-reported experience measure
PRO: Patient-reported outcome
PROM: Patient-reported outcome measure
SUS: System usability scale
VBHC: Value-based health care
Wi-Fi: Wireless fidelity
