# Supplementary material for: Rationale and development of an e-health application to deliver patient-centered care during treatment for recently diagnosed multiple myeloma patients: pilot study of the MM E-coach
Source: Pilot Feasibility Stud. 2023 May 20;9:85. doi: 10.1186/s40814-023-01307-0 (PMC10199287; doi:10.1186/s40814-023-01307-0)
Supplement: Supplementary file 1 — Additional file 1: Fig. S1. Mockup example of a medication overview for patients of one of the treatment schedules (Rd; lenalidomide/dexamethasone and co-medication). The text boxes next to the date display time by day or week; The columns represent medication type, medication dose, medication time and additional instructions. Fig. S2. Medication overview of one of the treatment schedules (KRd; carfilzomib/lenalidomide/dexamethasone and co-medication). The menu to the left is identical to figure 3; the columns to the right are comparable to figure S1. Fig. S3. Medication registration page. The menu to the left is comparable to figure 3. Each line represents one medication with information, for example acetylsalicylzuur translates to acetylsalicylic acid and the bold text to ‘dosing’, ‘aim’, ‘brand types’, ‘time of preference’, ‘side effects’, ‘additional remarks’. Fig. S4. Outpatient visit preparation questionnaire (not showing blank space). The menu to the left is comparable to figure 3 and marks ‘Outpatient clinic preparation’. The text contains an introduction asking patients what complaints or side effects they experience at that give moment and states that a patient can tick as many boxes as apply to that moment. Then every line represents one complaint, for example ‘Braken’ means ‘Vomiting’. Fig. S5. Periodic assessment, one question example. The menu to the left is comparable to figure 3 and marks ‘Periodic check’. The question translates to ‘How would you judge your health during the last week?’ and the answers range from ‘very bad’ to ‘excellent’. Fig. S6. Ad hoc complaint form completed, followed by advice. This displays the healthcare provide account, zooming in on one specific part of a questionnaire that was filled out by a patient. The second yellow alert line states the question ‘Do you experience vomiting for more than 24 hours’, as a follow-up question when the patient has ticked ‘vomiting’ in the list in figure S4. At the most right it states that [file 40814_2023_1307_MOESM1_ESM.zip › Figure S2R1.pdf]

SANACOACH

TE DOEN 1

Controleer uw medicijnen Start

IK WIL

Een beladvies Start

Een bericht sturen Start

Dag overzicht 04-09-2020

Medicijn	08:00	22:00	Kuur medicatie	Zo nodig
Acetylsalicylzuur	✖			
Allopurinol	✖			
Ciprofloxacin	✖			
Cotrimoxazol	✖			
Dexamethason	✖			
Fluconazol	✖			
Valaciclovir	✖			
Ciprofloxacin		✖		
Lenalidomide		✖		
Valaciclovir		✖		
Carfilzomib<br>*Carfilzomib, 20 mg/m2, Infusievloeistof, Infuus*			✖	
Rasburicase			✖	
Metoclopramide				✖
Metoclopramide				✖
Metoclopramide				✖

OK
