# Supplementary material for: Rationale and development of an e-health application to deliver patient-centered care during treatment for recently diagnosed multiple myeloma patients: pilot study of the MM E-coach
Source: Pilot Feasibility Stud. 2023 May 20;9:85. doi: 10.1186/s40814-023-01307-0 (PMC10199287; doi:10.1186/s40814-023-01307-0)
Supplement: Supplementary file 1 — Additional file 1: Fig. S1. Mockup example of a medication overview for patients of one of the treatment schedules (Rd; lenalidomide/dexamethasone and co-medication). The text boxes next to the date display time by day or week; The columns represent medication type, medication dose, medication time and additional instructions. Fig. S2. Medication overview of one of the treatment schedules (KRd; carfilzomib/lenalidomide/dexamethasone and co-medication). The menu to the left is identical to figure 3; the columns to the right are comparable to figure S1. Fig. S3. Medication registration page. The menu to the left is comparable to figure 3. Each line represents one medication with information, for example acetylsalicylzuur translates to acetylsalicylic acid and the bold text to ‘dosing’, ‘aim’, ‘brand types’, ‘time of preference’, ‘side effects’, ‘additional remarks’. Fig. S4. Outpatient visit preparation questionnaire (not showing blank space). The menu to the left is comparable to figure 3 and marks ‘Outpatient clinic preparation’. The text contains an introduction asking patients what complaints or side effects they experience at that give moment and states that a patient can tick as many boxes as apply to that moment. Then every line represents one complaint, for example ‘Braken’ means ‘Vomiting’. Fig. S5. Periodic assessment, one question example. The menu to the left is comparable to figure 3 and marks ‘Periodic check’. The question translates to ‘How would you judge your health during the last week?’ and the answers range from ‘very bad’ to ‘excellent’. Fig. S6. Ad hoc complaint form completed, followed by advice. This displays the healthcare provide account, zooming in on one specific part of a questionnaire that was filled out by a patient. The second yellow alert line states the question ‘Do you experience vomiting for more than 24 hours’, as a follow-up question when the patient has ticked ‘vomiting’ in the list in figure S4. At the most right it states that [file 40814_2023_1307_MOESM1_ESM.zip › Figure S4R1.pdf]

Voorbereiden spreekuur

11%

Van welke van de onderstaande klachten heeft u op dit moment last? U kunt meerdere klachten selecteren. Als u geen last heeft van onderstaande klachten, druk dan op Volgende.

Heeft u andere klachten dan hier beschreven en maakt u zich zorgen, stuur dan een bericht via deze SanaCoach (Ik wil: een bericht sturen) naar de regieverpleegkundige.

- [ ] Rillingen of koorts
- [ ] Bloedneus
- [ ] Bloedend wondje
- [ ] Bloed in uw ontlasting of urine, pikzwarte ontlasting
- [ ] Diarree
- [ ] Obstipatie (verstopping van de darmen)
- [ ] Huiduitslag
- [x] Braken
- [ ] Pijn
- [x] Misselijkheid
- [x] Kortademigheid, benauwdheid
- [ ] Aanhoudend hoesten
- [ ] Witte of pijnlijke plekjes in de mond
- [ ] Vaak kleine beetjes plassen of een branderig gevoel bij het plassen
- [ ] Tintelingen/gevoelloosheid in ledematen (voeten en/of handen)

VORIGE

VOLGENDE
