# Supplementary material for: Rationale and development of an e-health application to deliver patient-centered care during treatment for recently diagnosed multiple myeloma patients: pilot study of the MM E-coach
Source: Pilot Feasibility Stud. 2023 May 20;9:85. doi: 10.1186/s40814-023-01307-0 (PMC10199287; doi:10.1186/s40814-023-01307-0)
Supplement: Supplementary file 2 — Additional file 2: Table S1: Summary of qualitative evaluation. [file 40814_2023_1307_MOESM2_ESM.docx]

**Appendices**

Appendix A: Pilot study questionnaire for healthcare professionals. The questionnaire was originally used in Dutch language and this version is a non-validated translation.

The following statements are answered on a 5-point scale ranging from ‘completely agree’ to ‘completely disagree’:

1. I am confident about the development opportunities with regard to future application functionalities.
2. I feel like I contribute to the future of healthcare, by using this application.
3. Using the application supports me with my daily activities within the oncology centre.
4. Using the application makes the care process more efficient.
5. Using the application makes my work less monotomous.
6. I can acquire more insight for our patients, by using the application.
7. This application has an added value for me.

Appendix B: Example of an empty case form. This version is a non-validated translation for purpose of the readers’ understanding.

Case week: [x] date: [dd-mm-yyyy] brought by: [name professional]

Case description

| 1. Give a short description of the situation and the intervention that happened? |
| --- |
| 1. What went different here, compared to usual care? |
| 1. What is the estimated injury that was prevented, if any? |
| 1. What kind of (adjustments to) care delivery was made? |
| 1. Additional remarks? |
