# Supplementary material for: Rationale and development of an e-health application to deliver patient-centered care during treatment for recently diagnosed multiple myeloma patients: pilot study of the MM E-coach
Source: Pilot Feasibility Stud. 2023 May 20;9:85. doi: 10.1186/s40814-023-01307-0 (PMC10199287; doi:10.1186/s40814-023-01307-0)
Supplement: Supplementary file 3 — Additional file 3: Appendix A.Pilot study questionnaire for healthcare professionals. The questionnaire was originally used in Dutch language and thisversion is a non-validated translation.AppendixB.Example of an empty case form. This version is a non-validated translation for purpose of the readers’ understanding. [file 40814_2023_1307_MOESM3_ESM.docx]

Table S1: Summary of qualitative evaluation.

|  | Patient | Healthcare provider |
| --- | --- | --- |
| 1. Medication module | “Useful to see wheter I’ve taken my medication.”  “Useful to have a day overview.”  “Useful information about the medication.”  “Other medication besides for MM needs to be in there.”  “Medication should be directly in the e-coach when the hematologist prescribes it.”  “I would like a medication intake reminder.”  “My medication is not always entered correct.”  “Medication registration does not always work.”  “I would like be able to register >24u after medication intake.”  “MedApp is more flexible.”  “Sometimes I cannot register all medication at one moment and have to perform this for each medication separately.” | To enter medication manually is not safe.  The workflow now needs to be aligned to the manual input of the medication.  Manual input of the medication is time consuming.  The medication buttons do not show fields that you would intuitively expect. A week overview would be handy. |
| 2. Outpatient visit preparation | - | It provides insight into patients’ performance status and neuropathy well.  It works well, especially the blank space to inform us about important things that a patient would like to discuss. |
| 3. Periodic assessment | - | The current display is not clear; we suggest a dashboard. |
| 4. Ad hoc complaint | - | - |
| 5. Messaging service | “Getting into contact is flexible and accessible.”  “I appreciate contacting my hematologist directly.” | Quick signaling of problems.  Accessible getting into contact.  Easy to refer a patient without the need to find a moment for a phone call.  Messages are depicted chronological instead of sorted by message. |
| 6. Alerts | - | A urinary tract infection was noted and timely intervened.  A patient was admitted on our request for dyspnea following a notification.  Patient was timely provided with supportive medication for a side effect. |
| 7. Information | - | Maybe information movies would add to the app. |
| 8. Personal care plan | - | - |
|  | | |
| General | “Both on my iPad and my phone it works fine.” | “This is not easy to work with for every person.”  “I have to login every time again, it does not remember me.”  “The app crashes sometimes.”  Patients may like to switch modules on or off depending on their wishes. |

*Sentences between “ “ indicate quotations, the other sentences are derived from minutes or notes.*
